# A Further Inquiry Concerning Constitutional Irritation, and the Pathology of the Nervous System

**Published:** 1836-01-01

**Authors:** 


### A Further Inquiry concerning Constitutional Irritation, and the Pathology of the Nervous System. By *Benjamin Travers*, F.R.S. &c.

[Concluded from the last Number.]

Our readers will remember, or, if they do not remember, they will find, on referring to the last Number of this Journal, that the work of Mr. Travers is divided into two parts. The first is more essentially devoted to the elucidation of our author's notions on the nature of irritation ; while the second treats especially of the pathology of the nervous system.

The first part was analysed completely in our last Number. The second part is divided into five chapters, and of these we noticed the two first. The remaining three will, therefore, form the subject of the present article, which will terminate the consideration of the work.

The chapters in question are devoted to the investigation of the following points. The third chapter is on nervous affections, consequent upon lesion or alteration of texture of the nervous organ—the fourth chapter is on the operation and effects of poisons on the nervous system—and the fifth is a summary, with conclusions, pathological and practical.

### On Nervous Affections, consequent upon Lesion or Alteration of Texture of the Nervous Organ.

It is a fault, almost inseparable, perhaps, from profundity of views, that not only their precise signification, but the order, also, in which they are arranged, are difficult of appreciation by ordinary minds. Yet simplicity is no mean attribute of science, and clearness of expression is as requisite to him who aspires to instruct, as depth of ideas and soundness of reflection. Certainly our author does not possess the art, the happy art, of unfolding, in a plain and intelligible manner, his conceptions and conclusions. Long periods and involved parentheses perplex the reader, and the rugged page is travelled over with uncertain and with painful effort. We regret this, because, by diminishing the number of those who comprehend, it necessarily, also, diminishes the number of those who respect Mr. Travers' opinions.

The object of the present chapter is obviously to connect the organic lesions of the nervous system with the consequent alterations of its functions, or in other words, with the symptoms that those lesions manifest. This is an operation of analysis. The facts should first be collected—those naturally related should be classified—and these larger masses resolved by analytical deduction into their essential elements. Yet this obvious method is, unfortunately, not pursued, and we wander through the chapter, uncertain where each turn of the seeming maze may lead.

Mr. Travers commences, with justice, by observing that great obscurity hangs over the pathology of the nervous system, and that this obscurity is dependent, in a great degree, on the manner in which that system is mixed up with the different organs and tissues of the body. Let us put this in a clear light by a simple illustration. Angular motion is the function of the knee-joint. If such angular motion is impossible, we know that some structural change in the parts concerned in the production of that motion has occurred—we know that this must of necessity be so. But the functions of the nervous system not being so simple, alterations of those functions do not necessarily prove any special alteration of its structure. The function of the median nerve is to convey sense and motion to the palm of the hand. But, if sense and motion of that part are lost, that is not sufficient evidence of lesion of the nerve itself, because we find, from experience, that pressure on the spinal marrow or brain, with which the nerve is connected, will produce the same effect, nay, that some passing irritation of the ganglionic nerves, with which the median is still more indirectly in relation, may temporarily occasion the phenomenon. Thus the functions of the nervous system being exceedingly complex, the alterations of those functions must depend on various causes, and the appreciation of those causes must be a matter of excessive difficulty.

Mr. Travers' view of the condition of our knowledge is not flattering. He observes :—

"The brain plays so large a part in the diseases of other structures, that the symptoms of its proper diseases is difficult of recognition; and it is a more frequent problem, whether any organic change exists, or the disease is functional only, in the brain than in any other viscus. A principal source of the variety and obscurity of its pathological phenomena is doubtless the peculiarity of its circulation, which, notwithstanding the admirable provision of the venous sinuses, renders the balance more delicate and susceptible, and the effect of its disturbance of more importance than in any other organ, if we except the heart. In truth, there are instances of daily occurrence, in which the most attentive and sagacious observers are unable to decide whether the disease or injury is organic; and if so, what it is must be altogether conjectural. It may be very gratifying to the pride of science to suppose, that the presence or absence of this or that symptom has instructed us beyond all doubt as to what the mischief is or is not: but Nature is not so uniform and tractable at to be thus easily read—and the truth is, that all we can do in a large proportion of cases, is to wait upon the indications and developments that time affords, having carefully removed all obvious impediments in the way." 340.

He relates three cases in which the symptoms of apoplexy were presented; yet no alteration of the brain could be detected on examination after death. The first case is unsatisfactory, but the two succeeding are remarkable.

*Case.* One night in January, a lad was heard to fall from the mast-head of a vessel in the river, and was found lying upon the deck, with his head under a cask, perfectly insensible. He remained in that state, his pupils dilated, his fæces and urine passing involuntarily, breathing about eleven times in the minute, pulse 168. He was twice blooded, the pulse sinking each time under the operation; on the afternoon of the third day, he died without a struggle.

Not the least vestige of injury in the brain or spinal chord was discovered on inspection. The viscera of the thorax and abdomen were more turgid with blood than natural, but also perfectly sound.

Such a case as this must be deemed extremely rare—so rare, that few surgeons could have met its parallel. The patient had the symptoms of concussion, rather than compression. Could the bleeding in that state have had any thing to do with the result? If so, the absence of appreciable alterations would be intelligible.

The other case was that of a prize-fighter, who was taken off the ground insensible, and apparently apoplectic. He died in eight hours. No lesion of the brain was discoverable. We agree with Mr. Travers in his statement, that there are no cerebral symptoms, be they ever so severe, that have not been observed in cases that have finally done well. But we must in fairness state our belief that these are the exceptions, not the rule, and that our general state of information with reference to the connexion of symptoms and lesions of the brain, is far from being so disheartening as our author's remarks might lead the inexperienced practitioner to infer.

Mr. Travers enumerates the various organic lesions seen in the brain and in its membranes. The catalogue need not detain us. Nor need a similar catalogue of the alterations of the spinal chord, and of its membranes.

The following would appear to be the inferences drawn by our author from the pathological data in question:—

"Now if there be no detectible difference between the convulsion of the epileptic, and that produced by a spiculum of bone irritating the dura mater, between the coma of an apoplectic, and that consequent upon compression from fracture or extravasation: if a large proportion of cases of injury of the head recover from a degree of suspension or disturbance threatening the worst result, when the surgeon's experience and discretion restrain him from officious interference, it is evident that functional disturbance, separately considered, is not a criterion by which to judge either of the presence or nature of organic mischief. Much of it may really be indicative of a power of reaction, promising in such circumstances, as the natural consequence and expression of irritation of an organ sufficiently retaining its integrity to admit of recovery, which mechanical injury of any amount would have prevented. Continued insensibility furnishes the worst prognosis, and next to that, those temporary and partial illuminations which fade almost as soon as they appear. If the signs of commotion subside, we may infer that lesion is seldom if ever present. The recovery of mind, however gradual, if it be but progressive, is a most auspicious sign. On the other hand, the increasing failure of the mind, and the giving way of the organic and vital functions, as e. g. the loss of power of the alimentary canal; the frequent pauses and increasing labour of respiration; the small running indistinct pulse, and the oozing of the exhalant capillaries, are the fatal prognostics.

I should say, therefore, that organic change is distinguished by the assemblage and combination of the symptoms, by their permanency or frequent recurrence, and in place of the signs of restorative power, by the progressive failure, whether rapid or gradual, of the system of nutrition, and consequent increasing embarrassment of the vital functions, as the circulation and changes of the blood, the secretions, temperature, &c." 346.

We must confess that we do not see the force of the preceding reasoning Mr. Travers argues thus:—An apoplectic patient has coma, and a patient labouring under compression from fracture or extravasation, has coma too; therefore, functional disturbance of the brain, separately considered, is not a criterion of the presence or nature of organic mischief. The only way of testing these general laws, is trying their truth in a particular case. One function of the brain is the regulation of the motion of the voluntary muscles. A patient suddenly loses the power of motion on one side of his body. From experience we pronounce that some pressure is exerted on the opposite side of the brain, and ninety-nine times out of the hundred we are right. Now in this case, disordered function of the brain is clearly an indication of the presence of organic mischief. The axiom will not bear much handling. The greater part of what we know during life of the existence of organic lesions in internal organs, is through the medium of disordered functions. Whether any one disordered function is alone capable of furnishing the means of accurate diagnosis, must depend on circumstances. Of course, it is the combination of symptoms that in general enables us to detect the nature of disease; but in the instance we have mentioned, hemiplegia, that one functional disorder pointed out not only the existence, but the site too of the lesion.

Coma with stertor, we should say, is another symptom that points out pretty clearly pressure of some sort on the brain. It may perhaps be objected to this assertion, that patients die with this symptom, and yet no extravasation of blood nor serous effusion can be found. But reasoning might suggest to us, that congestion of the venous system of the brain would occasion pressure just as much as extravasation; and what is now known by the term of "simple sanguineous apoplexy," appears to be pressure of this description. We make these observations to shew, that general deductions in medicine require to be taken cum grano salis.

Mr. Travers proceeds to state, that organic lesion of the brain is never perfectly recovered from; some remnant of alteration of function always stays behind. "It is not," he says, "the actual amount of the injury, and its indirect interference with the functions of the brain, that occasions or maintains this state, but the effect of a shock which has so powerfully and permanently impressed the organ, as entirely to have modified its susceptibilities and habits of action, and 'turned the walking-stick into a crutch.'"

The idealists and the materialists have ever been opposed in medicine. We do not use the term materialist in the moral sense in which it is frequently employed, but simply as expressing one who looks for the causes of symptoms in material alterations of the corporeal structure, and is averse to admit the operation of agencies, the nature of which is founded on presumption. A materialist, for example, would take exception to Mr. Travers's hypothesis respecting the effects of what he calls a shock. He would cunningly ask what a shock is? A man falls with his head upon the floor, and the brain receives a shock; that shock, arising from a physical concussion and collision, has surely something material about it. We observe this:—that if the shock has a certain quantum of intensity, the patient suffers for a certain time, and then recovers;—if the injury is more severe, some tangible physical alteration in the brain, its membranes, or the cranium, is produced, and particular and more permanent symptoms ensue. The nature of the mode in which the shock is obtained, and the decisive symptoms that follow, render it probable, to say the least, that the shock itself is a minor degree of alteration of the finer structure of the brain. If this be true, and the arguments are greatly in its favour, our author's position would simply imply, that a slight physical alteration occasions effects that are permanent; while a more apparent, and necessarily more severe alteration, does *not* occasion permanent effects. In the following sentiment we agree with our ingenious author:—

"Structural changes in the nervous system, which do not directly affect life, derive their symptoms from the nature and degree of their interference with the proper functions of that system, and with those of the parts which it supplies; in one case spasm, in another paralysis, either of sensation or motion; in a third, neuralgia is set up; and these affections may be variously combined, of which either the cerebral, the cerebro-spinal, or the sympathetic system may be the seat."

Mr. Travers thinks that the alterations dependent on inflammatory action in nerves, result from the vessels of the membrane. No doubt, this is to the nervous substance what the periosteum is to the bone.

" Vascularity, thickening and induration, softening, deposit of lymph between the fibres, or between the sheath and the nerve, so as to separate or to displace the fibres centrally or laterally, severally or in one or more packets ; tumors and cysts of various character, so situated, the neurilemma forming the investment; the fibres terminating abruptly in a tumor above and below ; the fibres passing direct through the centre of a solid tumor without displacement or separation, and in both cases the neurilemma continuous and enclosing the tumor ; the ganglion or nervous knot, in its varieties of size and consistence, ulceration from pressure, or wound, or contiguous inflammation, and the absorption of the medulla (wasting) comprehend all the diseased appearances with which I am acquainted.

The tumors above-named are homogeneous, firm, or pulpy ; or cavities of abscesses, or cysts of solid contents in a state of disorganization. In the Hunterian collection is one specimen of a firm tumor embracing a nerve, and throwing off fibres on either side at right angles to the nerve. It presents an exact analogy to the osseous tumor which is formed in the cellular tissue connecting the periosteum with the bone, and I should think has a similar origin in the sub-cellular tissue of the neurilem."

Mr. Travers, after remarking that in a large proportion of cases of neuralgia we can detect no organic alterations as a cause, observes that, in the most severe and enduring cases, we do find either a change in the brain, or in the nerve at its origin or in its course, or an external irritant acting upon either.

Mr. Travers refers to sundry cases. Thus, tic douloureux of the branches of the fifth pair of nerves, and impairment or loss of power of the retina, have been induced by diseased teeth—cerebral disturbance is well known to be occasioned in children by dentition—Mr. T. has seen complete and permanent amaurosis from an abscess between the pterygoid muscles.—In Dr. Pemberton's case, tic douloureux in the left infra-orbital nerve was occasioned by a bony deposit in the falciform process of the dura mater, a little distance from the crista galli.—In a case communicated to Mr. Travers by Sir A. Cooper, and fully published in a late number of this Journal by Dr. James Johnson, epileptic fits were occasioned by a similar deposit in the falx, attended with thickening of the contiguous dura mater and arachnoid, and gelatinous-like alteration of the subjacent brain. The following case was communicated by Sir Benjamin Brodie.

*Case.* " Mr. ———, had laboured for many years under severe pain of the face, which had been regarded as the effect of tic douloureux. He had also suffered from epileptic fits. Latterly the former symptom had subsided, while the fits became more frequent and more severe. After one of these attacks he expired with symptoms of apoplexy. There had been, for some time previous to his death, ptosis of the left upper eye-lid, which made me suspect that the brain was affected at the part from which the third pair of nerves derives its origin.

*Examination.*—The internal surface of the cranium exhibited marks of increased vascularity. The impressions of the vessels were larger and more numerous than usual, and the foramina of the vessels more distinct—in some places also there were reddish discolorations, and the projections of the basis cranii were unusually prominent. The membranes of the brain were preternaturally vascular. The vessels of the dura mater large and numerous, the tunica arachnoides thickened, and at the upper and back part of the left hemisphere of the cerebrum, an adhesion had been formed between the tunica arachnoides and the dura mater of about an inch or 1¼ inch in diameter. The veins of the pia mater were unusually turgid.

The cut surfaces of the cerebrum exhibited numerous bloody specks indicating increased vascularity, and also a red mottled appearance in many places. The texture of the brain was remarkably soft, especially in the crura cerebri, fornix, and adjacent parts. The fibrous appearance of the brain was particularly distinct. The nerves of the fifth pair in the cavernous sinuses were carefully examined and seemed free from disease. No excess of fluid in the ventricles. The membranes of the spinal chord exhibited marks of increased vascularity, but the chord had the usual appearance.

The viscera of the thorax and abdomen were free from disease, except a partial adhesion of the pleura and a small brown interstitial deposit in the liver." 356.

It is evident that we are not yet sufficiently stocked with facts of this description to enable us to generalize. Indeed what we at present know, amounts to little more than this—that in a few cases of epilepsy and neuralgia we have found lesions in the brain or spinal marrow—that these lesions have been various—but that, in general, they amounted to what would rather constitute a source of irritation than of serious disorganization. A spiculum of bone, for example, has been found upon the dura mater, or a little adhesion of that membrane with the arachnoid—or partial softening of the substance of the brain or spinal chord—or a small morbid growth in their interior. What we want, we repeat, is an addition to our stock of facts—generalization would be premature at present.

Mr. Travers relates an instance of concussion of the spinal marrow.

*Case.* On Aug. 4. a plumber, æt. 33, fell from a height of thirty feet. He alighted on his feet, but then fell backwards with great force, and his sacrum came in contact with a large stone. He was unable to rise, although he had some motion of the lower limbs. On admission into the hospital, no signs of fracture were discoverable. At 4 P.M., he had excessive pain in the loins and legs, with total loss of motion—great depression—skin cold—pulse slow. Warm brandy and water given. 6 P.M. Re-action somewhat violent, exquisite morbid sensibility of the legs and feet; when touched slightly, pain of the most acute and lancinating character is produced. Pulse 120, hard and wiry, skin hot, great pain of head; C. c. lumb. reg. ad. ℥xvj.

10 P.M. The cupping relieved him for a short time, but now the local and general symptoms are more severe than before: the slightest motion, even that caused by walking up to the bed-side, brings on the most agonizing pain of the whole of the lower extremities, particularly on the posterior surface, and upon the bed-clothes being touched, he exclaims most piteously: head-ache violent: v. s. ad. ℥xvj. Liq. opii sed. ♏xxx. Calomel gr. iij. 4tis horis. His water was drawn by the catheter.

5th, 6 A.M. Has had no sleep; pain not quite so violent, and confined more particular to the back of the thighs, calves of the legs, and top of the feet; head-ache; pulse 120, sharp. Aperient draught. 12 o'clock. Bladder and sphincter ani paralysed; pain the same. Evening. Complains of pain in the lumbar region; pain in the legs again increased to the most excruciating degree; (to use his own words, when any one walks by his bed, it is just as if a number of razors were cutting him down to the bone;) pain more severe in the left than the right limb, which is colder to the touch; head-ache violent, skin hot, pulse irritable.

6th. Slept a little during the night, pain somewhat abated; countenance anxious, skin moist, pulse 112, full and soft. No aggravation of symptoms in the evening, as before; to increase the opiate to-night, and continue the calomel. On the 7th, the pain was diminished—the paralysis the same. In the evening he was able to move the toes a little. The pulse was 104, and soft—the mouth sore. Mercury omitted. From this time the improvement was not only progressive, but rapid. On the 8th of September he could walk with the assistance of crutches, and some weeks after leaving the hospital, he walked three miles with the assistance of a stick, his only inconvenience being a trembling of the legs, when fatigued.

This case is practically very instructive. The treatment was highly judicious. So soon as inflammatory symptoms came on, venesection, cupping, mercury, were the means resorted to. Such means are often necessary and sometimes highly useful after injuries of the spine—not only concussion, but even fractures of the vertebræ. The case too is physiologically interesting, exhibiting, as it does, the heightened sensibility of the parts supplied with nerves from the injured and subsequently inflamed portion of the spinal marrow.

A case is quoted from the practice of our author's colleague, Dr. Roots. It is this—

*Case.* A man was admitted in August, 1834, with total loss of motion, and partial loss of sensation of the lower extremities, and lived about a month. There was pain on pressure or percussion on one spot, viz. the 5th and 6th dorsal vertebræ, and on no other part of the spinal column. "This," says Dr. Roots, "I speak confidently, because I was astonished to find, at the post mortem examination, most extensive caries of nearly the whole of the bodies of the vertebræ below the 5th, where the disease commenced. Just opposite the 5th and 6th dorsal vertebræ, the whole of the spinal chord was softened to the consistence of thick cream *to the extent of five-eighths of an inch*, but not exceeding that;—the membranes were not appreciably thickened. I addressed my pupils on more than one occasion on the subject of the absence of pain on percussion, where there was such extensive disease of the bones."

We do not conceive cases of this description to be extremely rare. We have ourselves witnessed more than one instance, where extensive caries of the spine was attended with little or no pain on pressure and percussion. We recollect an instance in St. George's Hospital, where great destruction of the dorsal vertebræ was not accompanied with any appreciable tenderness, even on a careful examination.

Mr. Travers refers to the opinions lately promulgated with reference to many of the local pains in hysterical females—that they are dependent on irritation of some portion of the spinal marrow. He seems to attach much credit to these fancies. We cannot agree with him when he observes:—The painful arm after bleeding has been attributed to the puncture of a cutaneous nerve by the lancet, but the conclusion is gratuitous, fascial or venous inflammation, of which one or the other is present, being fully sufficient to explain the symptoms. We are not aware that any good surgeon does attribute the " painful arm" to puncture of a nerve *when* fascial or venous inflammation exists. It is when no other obvious cause does exist, that that cause is deemed to be puncture of the nerve. It is fascial inflammation, the term used unsuspiciously by Mr. Travers, that is understood most vaguely and erroneously. That fascial inflammation is usually nothing more nor less than inflammation of the cellular tissue above the fascia or below it.

Mr. Travers relates the particulars of two experiments, details some cases, and makes many observations on the consequences of injuries of nerves. For these we must refer our readers to the volume in which they are contained. The following case narrated by our author is, it is to be hoped, not common. It is that of a young female.

" Her own statement is, that a tumor following a blow from a fender upon the back of the hand, in 1828, led to her becoming a patient of Mr. Green, in St. Thomas's Hospital; that it was opened in several places without relief; that she was afterwards a patient of Mr. Vincent, in Bartholomew's Hospital, that three caustics were applied, and no relief obtained. She then again obtained admission at St. Thomas's, when Mr. Tyrrell amputated the hand two inches above the wrist. This was in November 1830; that the disease returning, a second amputation was performed by Mr. Langstaff, of Basinghall-street, three inches above the elbow, and that the diseased portions of the nerves were afterwards removed by that gentleman, by two incisions of the stump. Still unrelieved, she tried St. Bartholomew's a second time, as a patient of Mr. Earle, and from thence migrated eastward, and was the subject of another operation for excision of the nerve, performed by Mr. Luke of the London Hospital; that having other gentlemen's advice without benefit, and getting no ease night or day, she at length placed herself under Mr. Bransby Cooper, in Guy's, and had the stump removed at the shoulder-joint, in 1834, from which period she remained free from pain." 383.

We have heard or read of this case before, and our impression is that, the pain *has* returned since the date of our author's information. We say this is our impression. The truth is that operations are frequently gratuitous cruelty in these cases. The cause of the suffering is not in the part where it is felt--it is only *referred* to that. Its removal is not therefore the removal of the cause, and after operations have been again and again performed—after the patient has been put to great pain and to some risk—after he or she, for the sufferer is usually a female, has been mutilated secundum artem, she remains unrelieved of her sufferings, a memento at once of the weakness and the powers of art.

As an example of the injurious effects of operating upon *painful* tumors, Mr. Travers relates the following case from the practice of Sir Benjamin Brodie.

*Case.* The lady, the subject of it, had aneurism by anastomosis on the forehead. Sir B. Brodie tied the base of the aneurismal tumor with the effect of removing the disease for six years. Then a pulsating tumor again made its appearance, and was exquisitely painful. Sir B. Brodie tied its base again. This was done on the 9th of March. On the 11th, a little diffuse inflammation of the forehead supervened. On the 14th this was subsiding; but she complained of a little pain in the right forearm, and she was delirious, next day, the forearm was much swollen from the elbow to the wrist. The swollen part was tender, but there was little redness of the skin. There was neither inflammation nor swelling of the neck or arm, so that it was evident that there had been no extension of the inflammation to the extremity from the head and face. At 11, p. m. the whole forearm was in a gangrenous condition. Next day she died. No inspection of the body was permitted.

Now we doubt whether Mr. Travers can fairly cite this as an instance of the peculiar dangers attendant upon operations on painful tumors. Such consequences occasionally follow *any* operations, or *any* accidents. A man, for example, was admitted into St. George's Hospital with a simple scalp wound. Diffuse inflammation of the scalp occurred, and two or three incisions were made in it. He seemed to be doing well, when suddenly the right lower extremity fell into the state that was noticed in the upper limb in the last case, it mortified entirely, and the patient died. Sir B. Brodie operated on a woman in St. George's Hospital for scirrhus of the breast. So far from its being a painful tumor it was a remarkably indolent one. A few days after the operation, which had been succeeded by some suppuration in the immediate vicinity of the wound, the left lower extremity became gangrenous and the patient died.

The truth is, that these cases are analogous to the instances of secondary inflammation in distant parts after injuries and operations. From slight erythema on the skin, to extensive suppurations and to gangrene, we pass through all the phases of inflammatory action in cases of this description. It is not merely because the tumor operated on may be a painful one, that we meet with these sequelæ; their real cause must be sought in deeper and more obscure pathological conditions of the part or of the system.

Mr. Travers is disposed to admit too easily the direct interference of the nervous system. He observes for example:—

"The following case seems to show the direct tendency of injuries of nerves to produce that species of inflammation termed erysipelas, which if not especially under the nervous influence, is at least remarkable for its nearest affinities.

*Case.* In 1833 a man struck his leg violently against the points of a pair of scissors used for cutting tin. The wound was on the posterior and outer side of the leg, in its upper third, and passed round the fibula to the interosseous ligament in front. In a few days he was attacked with erysipelas of the limb and extensive sloughing of the cellular membrane in the vicinity of the wound, under which, in spite of treatment, he quickly sunk. On examination, the anterior tibial nerve was found three parts divided. There had not been the slightest symptom of spasmodic action or other nervous derangement. The preparation is preserved in the museum of St. Thomas's Hospital." 371.

Now such an injury, a punctured wound, is constantly found to give rise to diffuse inflammation and to erysipelas, quite independently of any lesion of a nerve. It is not, therefore, possible to single out that lesion when it does occur, and attribute in an especial manner to it, what we constantly observe without it.

Tumors of nerves may arise from neurilemmatous inflammation, occasioning deposition of lymph—or they may have a constitutional cause, and affect, or actually assume the true malignant character—or they may occur under a common form, which surgeons often see, and our author thus describes.

"Cases of small and isolated tumors of a gristly firmness, varying in size from a grain of wheat to a large pea, exquisitely painful, and requiring extirpation from that cause, are met with in the course of subcutaneous nerves. They are generally traceable to a blow or sprain. I have seen them in the breast, the thigh, the spermatic cord, the fingers, the instep, and various parts of the body. It is not always easy to determine if the tumor is in the nerve, or superjacent and adherent to it. Both these cases occur, and in the latter, I believe the removal of a portion of the nerve would be a safer practice than the simple extirpation of the adherent tumor. The latter proceeding, at least, I have twice found ineffectual in removing the pain, which is the gravamen of the disease." 381.

The most frequent cause, however, of the ganglionic tumor is amputation.

"It appears, then, that the ganglionic tumor occurs without wound, as 1st, in clusters upon the fibrillæ of a nerve; 2d, single or isolated upon a nerve subcutaneous or deep-seated, moveable, or adherent to contiguous parts; 3d, after section and re-section, as in amputation, in an irregular-shaped knot or knots, all the nerves of the limb, or on the same side of the limb, running into it or them, both trunks and branches, and so bound together at their termination.

In addition to this statement, I may add, that tumors of various structures lying upon and adherent to nerves, sometimes occasioning the neurilemmatous inflammation, and are equally productive of the peculiar pain which constitutes the disease, and that it is indispensable in such cases to remove a portion of the sound nerve above and below the diseased portion, to give the patient a chance of relief. 384.

The fifth chapter is devoted to the investigation of the operation of poisons on the nervous system. We do not think it necessary to advert to it more particularly. The sixth and last chapter is termed a summary, and contains "conclusions, pathological and practical."

The gist of the summary is a defence, and in many parts an eloquent defence, of our author's opinions on the agency and influence of the nervous system in the production of disease. He observes, and probably he is not far wrong, that functional diseases belong to the nervous system, and organic diseases to the vascular, either being capable of setting up the other.

"I admit," he says, "at its proper worth the force of the axiom 'Nil quod non demonstrandum,' and am alive to the prudent and prevailing jealousy of speculation as a substitute for reality, in the physical sciences inadmissible and in all unsatisfactory; but the animal movements are not, and never can be, subjected to mathematical adjustment, and unless we conclude that the actual operations of the nervous system are mere fictions, dreams like those which emanate from it, because we cannot unfold them, the argument drawn from their perversions and obliquities rests on the same foundation and is equally valid and tenable." 430.

*We* admit that the animal movements are not, and never can be, subjected to mathematical adjustment; but we do not think that, on that account, medicine should not be brought as near to an exact science as possible. We cannot shut our eyes to the obvious fact, that the great modern improvements, those mighty improvements productive of so much immediate benefit to suffering humanity, of so much more probable ultimate utility, we cannot, we say, shut our eyes to the obvious fact, that they have been achieved by the study of organic changes. There are many, no doubt, who are ultra-montane in their views, and hold morbid anatomy at a much higher price than it is worth. Yet we do not think we have yet obtained all that is to be obtained on the side of exactness, and we are sure that the way to effect the greatest possible amount of good, and to give to medicine all of demonstration that it can have, is to look with a very jealous eye on every thing approaching to hypothesis.

It would seem to be the opinion of some that the laws of functions and of functional alterations are hardly susceptible of strict analysis, and should not be tried by the test of induction. Such are not our sentiments. Induction may be well applied to the investigation of functional disorders. The results will not always be conclusive—again and again we shall be reminded how little we know and can ever know—but still what we *do* know will be satisfactory, and what we do *not* know will be manifested. The consciousness of ignorance is knowledge, and very valuable knowledge too, and that consciousness is frequently diminished, and sometimes altogether destroyed, by too ready an admission of reasoning avowedly not grounded upon demonstration.

Let us not be misconceived. We do not deny the agency, the influential agency of the nervous system. On former occasions we have raised our voices loudly against the mad attempt to smother a belief in it. But we really profess ourselves jealous of receiving very easily explanations founded on operations of that system, which are dubious in their nature and with difficulty understood. Mr. Travers then and ourselves are not the representatives of opposing principles—but the difference between us is simply one of plus and minus. Nay we think that on many points Mr. Travers is probably right, where a rigid criticism might plausibly expose the logical fallacy of reasonings necessarily built upon presumption. We think too that Mr. Travers is entitled to much credit, for boldly advocating principles which are not popular at present, but which he honestly believes should be opposed to the prevalent mania for ontologism.

In the following sentiments we most cordially coincide with Mr. Travers :—

"The history of any constitutional disease taken by itself would be of little use as a guide to the treatment of a patient, we must know the 'entourage', if I may so express it, how the system at large has been influenced by its previous state and ordinary habits, the natural temperament and the physical form, how far they have aided or kept back the development and are prognostic of its course, together with many other circumstances pertaining to the individual and the malady. If we want the character of a man's mind, we refer to his education and habits, and in the same way we must refer to the constitution if we would know its ailments. Now one of the chief points of such inquiry in a case of disease, is to ascertain whether the nervous or the vascular system predominates; and as regards the patient, whether he is an inflammatory or an irritable subject; than which no two constitutional characters are more strongly in contrast, or respectively more important in their influence. Nor are such inquiries of less weight in surgical maladies, even in casualties; they point as strongly to the nature of the constitutional disorder to be anticipated, as they explain that which has actually occurred, to inflammatory or to typhoid fever, to cerebral or hepatic derangement, to erysipelas or gangrenous inflammation, or tetanus, and even in the sequel, to scrofulous or carcinomatous action." 431.

We now take our leave of the able author. If we have differed from him with freedom, we have done so with respect. As a gentleman, we must esteem, as a man of ability we must admire him—if we have combated opinions which seemed to us to be of dubious accuracy, we have not lost sight of the possibility that the critic might be wrong, and the doctrine criticised right—and, if we have preferred our own conviction to a mere deference to the sentiments of Mr. Travers, we may say with Brutus—

It was not that we loved Cæsar less, but that we loved Rome more.

